# Emergency department visits among people with predementia highly predicts conversion to dementia

**DOI:** 10.1371/journal.pone.0270284

**Published:** 2022-06-24

**Authors:** Chia-Min Chung, Po-Chi Chan, Cheng-Yu Wei, Guang-Uei Hung, Ray-Chang Tzeng, Pai-Yi Chiu

**Affiliations:** 1 Department of Emergency Medicine, Show Chwan Memorial Hospital, Changhua, Taiwan; 2 Department of Neurology, Show Chwan Memorial Hospital, Changhua, Taiwan; 3 Department of Exercise and Health Promotion, College of Education, Chinese Culture University, Taipei, Taiwan; 4 Department of Nuclear Medicine, Chang Bing Show Chwan Memorial Hospital, Changhua, Taiwan; 5 Department of Neurology, Tainan Municipal Hospital, Tainan, Taiwan; 6 Department of Applied Mathematics, Tunghai University, Taichung, Taiwan; University of Bologna, ITALY

## Abstract

Emergency department visits (EDV) are common among older adults with and without dementia. The risk factors and demands of EDVs for people with dementia have been well studied; however, the association between EDVs and conversion to dementia among people with predementia has not been thoroughly explored. To study the predictive value of EDVs in predementia’s progression to dementia. The baseline predementia cohort registered from September 2015 to August 2017, with longitudinal follow-up in the History-based Artificial Intelligent Clinical Dementia Diagnostic System database, was retrospectively analyzed. The rates of conversion among the different EDVs were compared. Multivariate logistic regression and Cox proportional hazards analyses were applied to study the influence of EDVs on progression. Age, education, sex, neuropsychological tests, activities of daily living, neuropsychiatric symptoms, parkinsonism, and multiple vascular risk factors were adjusted for. A total of 512 participants were analyzed, including 339 (66.2%) non-converters and 173 (33.8%) converters with a mean follow-up of 3.3 (range 0.4–6.1) and 2.8 (range 0.5–5.9) years, respectively. Compared to people without EDV (EDV 0), the hazard ratios for conversion to dementia were 3.6, 5.9, and 6.9 in those with EDV once (EDV 1), twice (EDV 2), and more than twice (EDV >2), respectively. In addition, older age, lower education, poorer cognition, poorer ADL performance, and longer follow-up periods also increased the conversion rates. EDVs in the predementia stages highly predict progression to dementia. Therefore, a sound public health as well as primary healthcare system that provide strategies for better management of mental and physical condition might help prevention of EDVs among older people in the predementia stages.

## Introduction

Older adults visit emergency departments more often than younger people [1]. However, people with dementia [1, 2] visit emergency departments less frequently than the general older population [2]. Common etiologies for emergency department visits (EDVs) among patients with or without dementia are similar, including pneumonia, urinary tract infections, injuries or falls, congestive heart failure, cardiovascular disease, cerebrovascular disease, delirium, and pain [1, 3–6]. Compared to patients without dementia, people with dementia have longer stays and higher rates of hospitalization when they visit the emergency department [2]. Delirium is a precipitating factor that is highly associated with older people becoming demented and it is also an independent predictor of increased six-month mortality, especially when older people visit emergency rooms [6, 7]. Studies focusing on the frequency, association, and etiologies of EDVs among people with or without dementia are robust [1–7]; however, few studies have addressed these EDV factors in the predementia stages. Most studies have revealed that people with mild cognitive impairment (MCI) have a higher rate of EDVs than those without cognitive impairment [8, 9]. To the best of our knowledge, studies that can provide evidence for EDVs contributing to progression from MCI to dementia are lacking.

Understanding the prediction or prevention factors for predementia conversion to dementia may help prevent disease progression [10–14]. Candidate factors, including clinical information [10–12], liquid biomarkers [15, 16], and imaging biomarkers [17, 18], have been widely studied in the past few decades. Among these predictors, clinical information, including data on personal history, medical history, and neuropsychological performance acquired from participants themselves or their informants, is direct and cost-effective and can be widely used in clinical settings [19, 20]. Therefore, we aimed to investigate possible clinical factors for progression to dementia among people with predementia, emphasizing EVD. Based on evidence for raising the possibility of cognitive decline or functional decline after EDV in older people, in the current study, we initially investigated the factors associated with EDV among individuals in predementia stages. We expected that the common etiologies for people with predementia visiting emergency departments would be similar to those of normal older people visiting emergency departments. Subsequently, we determined the contribution of the frequency of EDVs to the conversion of dementia using longitudinal follow-up analysis. We proposed that more frequent EDVs would correlate with increased conversion to dementia based on the assumption that common etiologies for older people visiting emergency departments are also risk factors for older people developing dementia. Furthermore, we expected to provide useful clinical information for healthcare policies or education that increasing EDVs might also increase conversion to dementia, a sound public health as well as primary healthcare system that provide strategies for better management of mental and physical condition might help prevention of EDVs among older people in the predementia stages.

## Methods and participants

This was a retrospective longitudinal follow-up study that used data from the History-based Artificial Intelligent Clinical Dementia Diagnostic System (HAICDDS) project, which is currently being applied in three centers in Taiwan [20–22]. This project consecutively registers cognitively unimpaired people and individuals with cognitive impairment (CI) or dementia. During registration, all participants and their informants were interviewed by well-trained neuropsychologists and asked to complete a detailed medical history as well as neuropsychological, neurobehavioral, and activities of daily living (ADL) surveys. The detailed medical history included personal history, medical history, and family history. In addition to the Clinical Dementia Rating (CDR) scale [23] neuropsychological assessments, including the Cognitive Abilities Screening Instrument (CASI) [24], Montreal Cognitive Assessment (MoCA) [25], History-based Artificial Intelligent Activities of Daily Living (HAIADL) scale [26], and 12-item Neuropsychiatric Inventory (NPI) [27], are used to determine and trace the severity of CI or dementia. The CASI, which is composed of 9 cognitive domains, was popularly applied in Taiwan and many other countries using different languages [24]. The HAIADL, which was modified from Lawton and Brody’s IADL and BI (Barthel Index), composed of 15 questions and can be applied in individuals with ADL impairment due to cognitive disorders for the determination of severity of CI stages [26]. Diagnosis of all participants were made in a consensus meeting that composed of experienced neurologists, neuropsychologists, and radiologists. In this study, a cohort of predementia stages, including subjective cognitive decline (SCD) and MCI, with longitudinal follow-up data were selected and analyzed.

### Diagnosis of predementia stages including SCD and MCI or dementia in the HAICDDS database

A diagnosis of SCD includes a global CDR score of 0 in conjunction with subjective reports of cognitive decline. The CASI for SCD should be in the non-demented range after adjusting for age, sex, and education [24]. MCI was diagnosed according to the criteria proposed by Petersen et al. [28]. It is operationally defined as a change in cognition with impairment in cognitive tests in the CASI or MoCA, but without evidence of impairment in social or occupational functioning, with a global CDR score of 0.5 and a sum of boxes of the CDR (CDR-SB) <4.5 [29, 30]. The cutoff scores for MCI using the CASI should be in the non-demented range after adjusting for age and education [24]. Dementia was diagnosed according to the criteria for dementia proposed by the National Institute on Aging and Alzheimer’s Association (NIA-AA) [31]. Participants with impairments in two or more cognitive domains and a decline in daily functions, with a global CDR score ≥0.5 plus a CDR-SB score ≥4.5, are considered to have dementia. An HAIADL score >8 was used for the operational diagnosis of functional impairment [26]. CASI was used to test cognitive performance. The cutoff score should be in the demented range after adjusting for age, sex, and education [24]. Participants with delirium and without previous diagnosis of CI were excluded in the study.

### Definition of conversion

The baseline for both converters and non-converters was the first assessment. Each follow-up assessment was a checkpoint. The conversion is made after a complete assessment that fulfills the cutoff scores for dementia, defined as CDR ≥1 or CDR-SB ≥4.5. The CASI score should be lower than the cutoff scores after adjusting for age and education [28]. In addition, the CDR-SB, CASI, and HAIADL scores should deteriorate at later checkpoints compared to those at the baseline.

### Study procedure

**Fig 1:**
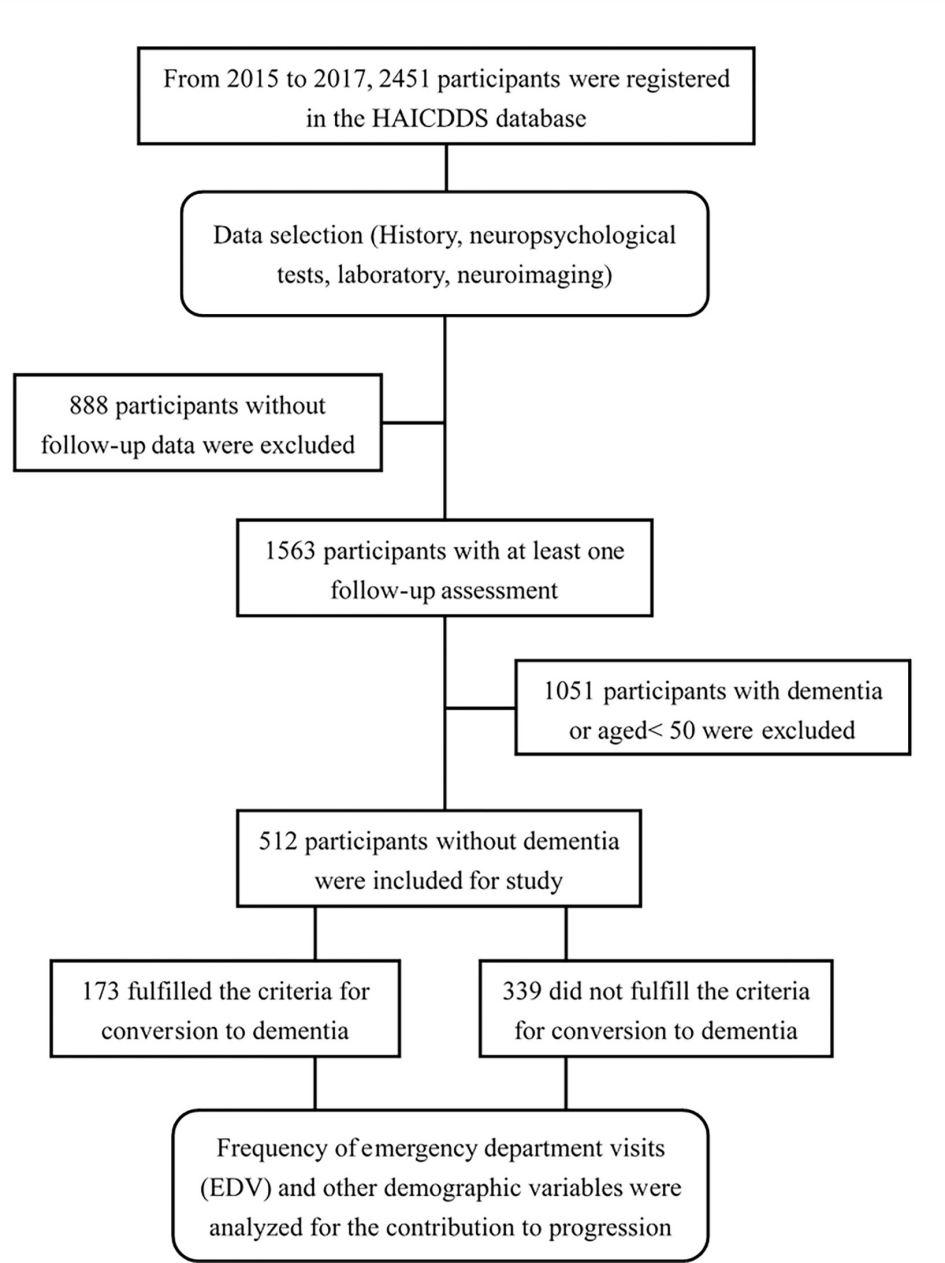
Flow chart for participant selection.

SCD and MCI cohorts registered from September 2015 to August 2017 with at least one follow-up assessment were studied. The following variables were analyzed: 1) demographic data including age, sex, education, follow-up period, history of cerebrovascular disease, parkinsonism, hypertension, diabetes, dyslipidemia, carotid artery disease, arrhythmia, and congestive heart failure; and 2) the CDR-SB and neuropsychological tests including CASI, MoCA, HAIADL, and 12-item NPI. Converters and non-converters were determined based on the demographic and neuropsychological variables analyzed. The common etiologies of EDVs were summarized. The frequencies of conversion and hazard ratios (HRs) according to different EDV rates were compared. The detailed procedure is shown in Fig 1.

### Statistics

The Chinese version of SPSS 22.0 for Windows (IBM SPSS Statistics, IBM Corp., Armonk, NY, USA) was used for statistical analyses. Age, education, follow-up year, and CDR-SB, CASI, MoCA, IADL, and NPI scores were analyzed using independent t-tests. Sex, CDR score, history of cerebrovascular disease, parkinsonism, hypertension, diabetes, dyslipidemia, carotid artery disease, arrhythmia, and congestive heart failure were analyzed using the chi-square test. Etiologies for people with predementia who visited the emergency department were summarized. The percentage frequency of conversion according to EDVs were summarized and a multiple logistic regression analysis was adopted to investigate the contribution of EDVs of the predementia participants to progression. The Cox proportional hazards regression was also adopted to investigate the contribution of EDVs among predementia participants to conversion to dementia. Hazard ratios were adjusted for age, sex, education, cognition (CASI), activities of daily living (HAIADL), neuropsychiatric symptoms (NPI), cerebrovascular disease, parkinsonism, diabetes, hypertension, dyslipidemia, coronary artery diseases, arrhythmia, and congestive heart failure. The significance level was set at *p* < .05 for all hypothesis tests.

### Ethical consideration

The participants were selected from the HAICDDS database of the Show Chwan Healthcare System. The study design was retrospective and the data were analyzed anonymously. The Committee for Medical Research Ethics of Show Chwan Memorial Hospital reviewed the project and the Data Inspectorate approved the study (IRB No: IRB1081006). The requirement of patient informed consent was waived by the ethics committee, as this study involved a retrospective medical record analysis.

## Results

A total of 512 participants were analyzed, including 339 (66.2%) non-converters and 173 (33.8%) converters with a mean follow-up of 3.3 (range 0.4–6.1) and 2.8 (range 0.5–5.9) years, respectively. Among them, 275, 107, 67, and 63 had 0, 1, 2, and >2 visits to the emergency department, respectively. In this population, 46.3% of participants had at least one EDV. The comparison of demographic variables between the non-converter and converter groups without adjustment revealed significant differences in age (*p* < .001), follow-up years (*p* = .001), EDV (*p* < .001), CDR score (*p* = .001), CDR-SB score (*p* < .001), CASI score (*p* < .001), MoCA score (*p* < .001), HAIADL score (*p* < .001), and diabetes (*p* = .022) (Table 1).

**Table 1:** Comparison of demographic data between non-converter and converter groups with CDR< 1.

	Non-converters	Converters	*p*-value
	Mean (SD; range)	Mean (SD, range)	
**N**	339	173	
**Age, year**	70.2 (8.4; 50–89)	74.8 (8.1; 53–91)	< 0.001
**Sex, female, N (%)**	190 (56)	94 (54)	NS
**Education, year**	6.0 (4.6; 0–18)	5.3 (4.2; 0–16)	NS
**Follow-up, year**	3.3 (1.3; 0.4–6.1)	2.8 (1.5; 0.5–5.9)	0.001
**ED visit**	0.8 (1.5; 0–10)	1.8 (2.3; 0–18)	< 0.001
**CDR, 0/0.5, N**	39/300	5/168	0.001
**CDR-SB**	1.5 (1.1; 0–4.0)	2.5 (1.2; 0–4.0)	< 0.001
**CASI**	79.8 (11.7; 50–100)	72.1 (11.7; 50–93)	< 0.001
**MoCA**	19.2 (6.3; 7–30)	14.7 (5.4; 7–27)	< 0.001
**HAIADL**	3.0 (2.0; 0–11)	4.7 (2.3; 0–12)	< 0.001
**NPI**	4.8 (6.7; 0–38)	5.5 (7.1; 0–36)	NS
**Cerebrovascular disease, N (%)**	51 (15)	35 (20)	NS
**Parkinsonism, N (%)**	71 (21)	40 (23)	NS
**Hypertension, N (%)**	162 (48)	90 (52)	NS
**Diabetes, N (%)**	71 (21)	52 (30)	0.022
**Dyslipidemia, N (%)**	81 (24)	32 (19)	NS
**Carotid artery disease, N (%)**	30 (9)	15 (9)	NS
**Arhythmias, N (%)**	23 (7)	14 (8)	NS
**Congestive heart failure, N (%)**	19 (6)	16 (9)	NS

Abbreviations: ED visit, mean frequency of emergency department visit; CDR, Clinical Dementia Rating scale; N, number; SD, Standard deviation; NS, non-significance; CDR-SB, sum of boxes of the CDR; CASI, Cognitive Abilities Screening Instrument; MoCA, Montreal Cognitive Assessment; HAIADL, History-based Artificial Intelligence Activities of Daily Living; NPI, Neuropsychiatric Inventory.

Etiologies for predementia patients who visited emergency departments were dizziness or headaches (16.8%), infection or sepsis (16.8%), acute abdomen or other gastrointestinal discomfort (10.6%), falls (6.2%), cardiovascular disease (6.2%), cerebrovascular disease (5.5%), traffic accidents or other traumas (5.5%), dyspnea (5.1%), head injuries or concussion (4.4%), delirium or disturbance of consciousness (4.4%), fractures (2.9%), seizures (1.8%), hypoglycemia or hyperglycemia (1.5%), urine retention (1.1%), fatigue or malaise (1.1%), and others (10.1%). Among all etiologies, those most related to CNS disorders, including dizziness or headaches, cerebrovascular disease, head injuries or concussion, delirium or disturbance of consciousness, or seizures, and accounted for 32.9% of EVDs. Therefore, CNS etiologies accounted for approximately one-third of cases and were the most common etiologies for predementia people who visited the emergency department in this study.

Baseline CDR-SB among EDV 0 (1.7±1.2), EDV 1 (2.0±1.3), EDV 2 (1.8±1.2), and EDV >2 (2.0±1.2) revealed no significant difference (*f* = 2.047; *p* = 0.106). Baseline CASI among EDV 0 (77.7±11.9), EDV 1 (76.4±13.2), EDV 2 (77.3±11.8), and EDV >2 (75.8±13.0) revealed no significant difference (*f* = 0.582; *p* = 0.627). Baseline HAIADL scores for EDV 0 (3.4±2.1), EDV 1 (3.9±2.6), EDV 2 (3.6±2.2), and EDV >2 (3.7±2.2) revealed no significant difference (*f* = 1.783; *p* = 0.149). Baseline NPI among EDV 0 (4.6±6.9), EDV 1 (5.9±7.6), EDV 2 (3.9±5.7), and EDV >2 (6.2±7.2) revealed no significant difference (*f* = 2.168; *p* = 0.091). The mean change from the baseline (MCB) of CDR-SB among EDV 0 (3.7±5.3), EDV 1 (4.7±5.0), EDV 2 (4.1±5.6), and EDV >2 (6.2±5.6) revealed significant difference (*f* = 4.043; *p* = 0.007). The MCB of CASI among EDV 0 (-18.5±27.7), EDV 1 (-20.6±26.5), EDV 2 (-18.1±27.7), and EDV >2 (-27.7±29.1) revealed no significant difference (*f* = 1.998; *p* = 0.113). The MCB of HAIADL among EDV 0 (7.2±9.3), EDV 1 (9.4±9.1), EDV 2 (8.9±10.0), and EDV >2 (12.2±9.6) revealed significant difference (*f* = 5.142; *p* = 0.002). The MCB of NPI among EDV 0 (2.4±12.1), EDV 1 (1.7±11.7), EDV 2 (4.4±10.2), and EDV >2 (3.0±11.9) revealed no significant difference (*f* = 0.766; *p* = 0.513). (S1 Table)

**Fig 2:**
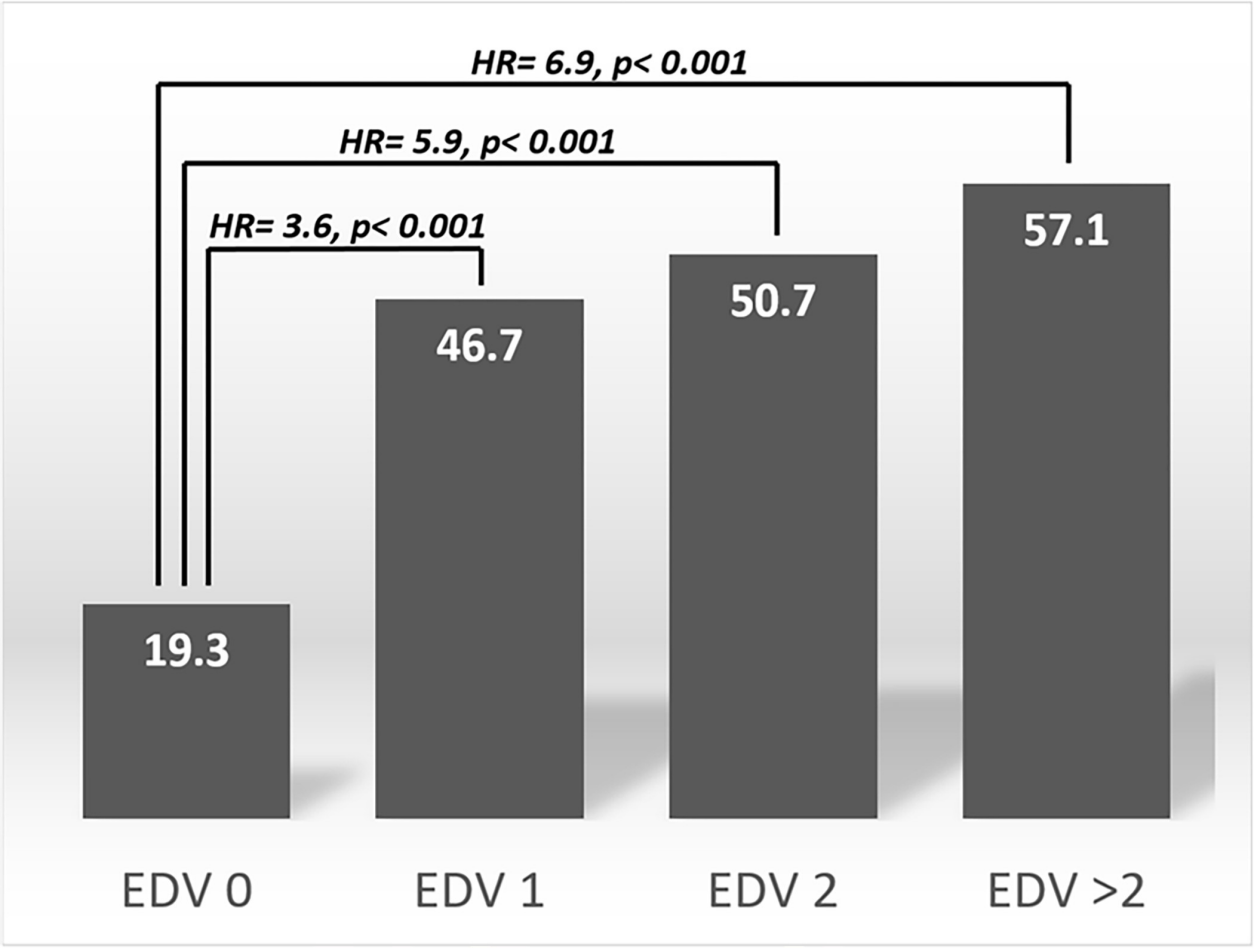
Percentage frequency of conversion according to emergency department visits (EDV). Multiple logistic regression analysis was adopted for investigating the contribution of EDV to the predementia participants’ conversion to dementia. Hazard ratios (HR) were adjusted for age, sex, education, cognition (CASI), activities of daily living (HAIADL), neuropsychiatric symptoms (NPI), cerebrovascular disease, parkinsonism, diabetes, hypertension, dyslipidemia, and coronary artery disease. Compared to EDV 0, HR were 3.6, 5.9, and 6.9 in EDV 1, EDV 2, and EDV >2, respectively.

Fig 2 demonstrates that the percentage frequency of conversion according to EDVs was 19.3%, 46.7%, 50.7%, and 57.1% for EDV 0, EDV 1, ECD 2, and EDV >2, respectively. Multiple logistic regression analysis was used to investigate the contribution of the participants’ EDVs to the conversion from predementia to dementia. Compared to those who did not visit the emergency department (EDV 0), HRs were 3.6, 5.9, and 6.9 in EDV 1, EDV 2, and EDV >2, respectively, after adjusting for all demographic and clinical variables (Table 2).

**Table 2:** Multiple logistic regression analysis was adopted for investigating the contribution of EDV and other variables of the predemented participants to convert to dementia. Hazzard ratios (HR) were adjusted for age, sex, education, follow-up year, CASI, HAIADL, NPI, cerebrovascular diseases, parkinsonism, diabetes, hypertension, dyslipidemia, coronary artery diseases, arrhythmia, and congestive heart failure.

Variables	B	Wald	Sig	Exp	95% CI for Exp
**EDV**					
**0**	0				
**1**	1.269	18.845	< 0.001	3.558	2.006–6.312
**2**	1.774	25.116	< 0.001	5.892	2.984–11632
**>2**	1.932	30.344	< 0.001	6.904	3.472–13.729
**Age**	0.048	9.267	0.002	1.049	1.017–1.082
**Sex**	0.185	0.531	0.466	0.831	0.505–1.367
**Education**	0.079	5.083	0.024	1.082	1.010–1.159
**Follow-up year**	-0.258	9.931	0.002	0.772	0.658–0.907
**CASI**	-0.048	13.339	< 0.001	0.953	0.929–0.978
**HAIADL**	0.298	26.088	< 0.001	1.347	1.202–1.510
**NPI**	0.002	0.010	0.922	1.002	0.969–1.035
**cerebrovascular diseases**	-0.162	0.270	0.603	0.851	0.462–1.565
**parkinsonism**	-0.140	0.248	0.619	1.150	0.663–1.998
**diabetes**	-0.224	0.656	0.418	0.799	0.465–1.375
**hypertension**	-0.147	0.380	0.538	0.863	0.540–1.378
**dyslipidemia**	0.156	0.293	0.588	1.169	0.664–2.060
**coronary artery diseases**	0.405	0.939	0.333	1.500	0.661–3.403
**arrhythmia**	-0.509	1.340	0.247	0.601	0.254–1.423
**congestive heart failure**	-0.380	0.679	0.410	0.684	0.277–1.689

Abbreviations: EDV, emergency department visits; CASI, Cognitive Abilities Screening Instrument; HAIADL, History-based Artificial Intelligence Activities of Daily Living; NPI, Neuropsychiatric Inventory.

**Fig 3:**
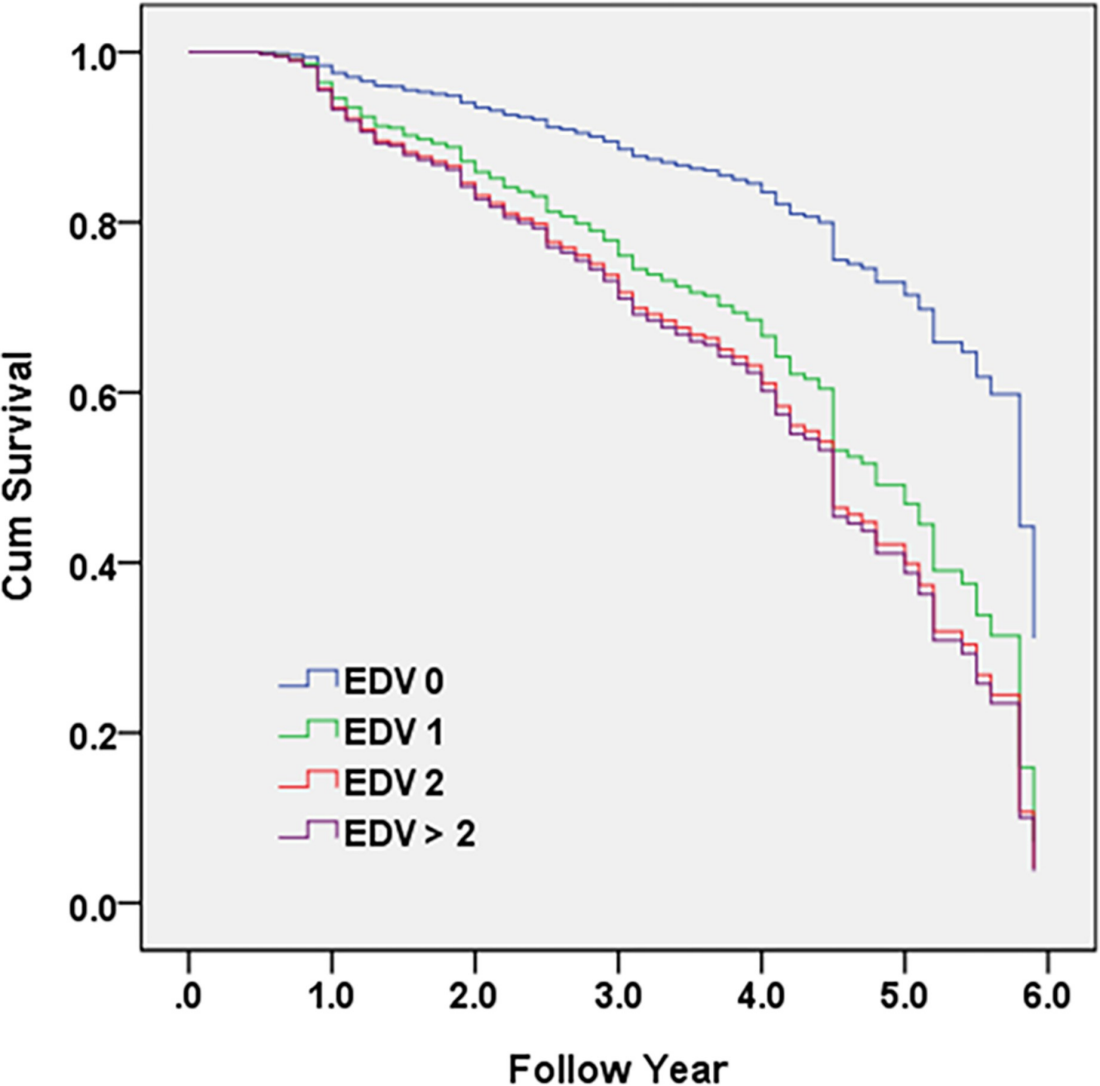
Cox proportional hazards regression was adopted to investigate the contribution of emergency department visits (EDV) among predementia participants to conversion to dementia. Hazard ratios were adjusted for age, sex, education, cognition (CASI), activities of daily living (HAIADL), neuropsychiatric symptoms (NPI), cerebrovascular disease, parkinsonism, diabetes, hypertension, dyslipidemia, and coronary artery disease.

Fig 3 demonstrates that Cox proportional hazards regression was adopted to investigate the contribution of participants’ EDVs to conversion from predementia to dementia. Hazard ratios were adjusted for age, sex, education, cognition (CASI), activities of daily living (HAIADL), neuropsychiatric symptoms (NPI), cerebrovascular diseases, parkinsonism, diabetes, hypertension, dyslipidemia, and coronary artery disease. Compared to those who did not visit the emergency department (EDV 0), HRs were 2.3, 2.7, and 2.8 in EDV 1, EDV 2, and EDV >2, respectively, after adjusting for all demographic and clinical variables.

## Discussion

Recently, researchers have put considerable effort into developing or discovering practical tools for tracing the deterioration of cognition or daily functioning in the predementia stages. In this study, we presumed that people who need to visit the ER may have more precipitating risk factors or comorbidities, leading to a faster decline in mental function. Following this ideal, we proposed a positive predictive value of EDVs by studying their contribution to conversion among people in the predementia stages. To the best of our knowledge, this study is the first longitudinal follow-up study that addresses the prevalence and contribution of EDVs among predementia people to conversion to dementia.

Several important findings from this study deserve attention. First, 46.3% of our predementia population had at least one EDV, indicating a higher prevalence and demand for EDVs in predementia compared to people with dementia [2]. Common etiologies among predementia people visiting emergency departments are not completely consistent with those among the general older population. Previous studies focusing on etiologies for general older adults or people with dementia who visited the emergency department showed that infectious disorders, including pneumonia and urinary tract infections, injuries, or falls were most common [1–6]. For example, in Gruneir’s review, injuries (33.1%), chest pain (4.9%), congestive heart failure (2.9%), and abdominal pain (2.8%) are the most common complaints [1]. In Lin’s study, except for dementia (7.7%), infectious disorders, including pneumonia (8.8%) and urinary tract infections (6.7%), were the most common complaints [2]. However, in this study, etiologies related to CNS disorders such as dizziness/headaches (16.8%), cerebrovascular disease (5.5%), head injuries/concussion (4.4%), delirium/disturbance of consciousness (4.4%), or seizures (4.4%) were found more frequently among predementia patients and accounted for a total of 32.9% in our study. This finding is not completely consistent with previous studies and the prevalence of CNS disorders in individuals with predementia visiting emergency departments deserves attention. In addition, EDVs due to infection or sepsis were also high in this study (16.8%), which is consistent with previous studies.

Second, comparison of the demographic characteristics between converters and non-converters revealed that converters were older (*p* < .001) and had higher CDR scores (*p* = .001), higher CDR-SB scores (*p* < .001), poorer cognitive performance according to CASI (*p* < 0.001) and MoCA (*p* < .001), poorer ADL functioning according to HAIADL (*p* < .001), and a higher prevalence of diabetes (*p* = .022). The findings that older age, higher CDR/CDR-SB, and poorer cognitive functions contribute to conversion to dementia were consistent with previous studies on demographic and clinical characteristics [32–35]. However, the association between ADL and progression to dementia has been explored less in previous studies, probably because unimpaired ADL is essential to diagnosis in the non-demented stages; however, researchers also found minimal to mild impairment in ADL in MCI [36–38]. Clarifying the association between ADL in predementia individuals and their conversion to dementia is warranted and deserves further investigation.

Furthermore, the HRs for EDV contribution to dementia were 3.6, 5.9, and 6.9 in EDV 1, EDV 2, and EDV >2, respectively, according to multiple logistic regression analysis (Table 2); these were 2.3, 2.7, and 2.8 in EDV 1, EDV 2, and EDV >2, respectively, according to Cox proportional hazards regression. Both statistical analysis methods confirmed the contribution of EDVs to conversion to dementia among the predementia population. In the perspective of conversion to dementia, we have not only adjusted cognitive and neuropsychiatric factors, but also adjusted the risk factors (CVA, parkinsonism, diabetes, and CAD) that might contribute to progression of SCD/MCI to dementia. We have also adjusted performance of daily functions including physical and motor functions using the HAIADL scale. In this study, we provided robust evidence of the highly predictive value of EDVs for the progression of dementia in people with predementia.

Finally, we considered determination of conversion to be the most difficult part for clinical or epidemiological studies that addressed conversion from non-dementia to dementia. Previous longitudinal studies have used cognitive screening tests and researchers’ diagnosis of incident dementia without a definite diagnosis of conversion and reversion [39–41]. Instead, for the determination of conversion, we have set up relatively strict criteria that use a diagnostic battery combined with multiple aspects for the diagnosis of incident dementia. Conversion is defined as deterioration on clinical assessment, including the CDR-SB, CASI, MoCA, and HAIADL, without any return to better performance or function at the turning point assessments as well as the following assessments. The conversion rate was much higher, even with only one EDV (46.7%), compared to those who had never visited the emergency department (19.7%). Therefore, EDVs might be a good candidate to provide a highly predictive value. EDVs in the predementia stages highly predict progression to dementia. Therefore, a sound public health as well as primary healthcare system that provide strategies for better management of mental and physical condition might help prevention of EDVs among older people in the predementia stages.

There are several limitations to this study that need to be addressed. First, few of our participants have done more determinative biomarkers, such as genetic study, amyloid plaque imaging, cerebrospinal fluid biomarkers, or pathological studies, the diagnosis of severity of cognitive impairment and conversion were based mainly on clinical criteria and assessment, supplemented with neuroimaging study and regular laboratory tests for dementia. Second, this study was conducted at three centers in Taiwan. Further studies that include more centers to investigate the effectiveness of EDV for tracing conversion or reversion in predementia stages are warranted. Third, not all participants were followed up for an equal duration; the follow-up period ranged from 0.3 to 5.5 years. Third, this was a retrospective longitudinal follow-up study; the design was not preplanned and the data acquired might not be precise or predetermined. Finally, for the determination of conversion in this study, a diagnostic battery combined with multiple aspects for the diagnosis of incident dementia was applied. Those without any return to better performance or function at the turning point assessments as well as the following assessments were regarded as conversion. Concept for the determination of conversion used in this study is based on the evidence of the high prevalence of fluctuating cognitive function or ADL functions in non-dementia stages. In addition, the diagnostic stages may also be unstable in CDR 0.5 including MCI or very mild dementia. Further studies to verify the clinical applicability of the concept determining conversion is this study is warranted.

In conclusion, EDVs in the predementia stages highly predicts progression to dementia. Therefore, health care policies and education, including how to prevent EDVs among older people, are warranted.

## Supporting information

S1 Table Comparison of demographical data between the non-converter and converter groups of the predemented participants. (DOCX)

## References

[1] Gruneir A, Silver MJ, Rochon PA. Emergency department use by older adults: a literature review on trends, appropriateness, and consequences of unmet health care needs. *Medical Care Research and Review*. 2011;68(2):131–155. doi: 10.1177/1077558710379422 20829235

[2] Lin PC, Lin LC, Hsieh HF, Chen YM, Chou PL, Li CH. Primary diagnoses and outcomes of emergency department visits in older people with dementia: a hospital-based retrospective study in Taiwan. International psychogeriatrics, 2020;32(1):97–104. doi: 10.1017/S1041610219000395 31030703

[3] LaMantia MA, Stump TE, Messina FC, Miller DK, Callahan CM. Emergency department use among older adults with dementia. *Alzheimer disease and associated disorders*. 2016;30(1):35. doi: 10.1097/WAD.0000000000000118 26523710 PMC4764430

[4] Samaras N, Chevalley T, Samaras D, Gold G. Older patients in the emergency department: a review. *Annals of emergency medicine*. 2010;56(3):261–269. doi: 10.1016/j.annemergmed.2010.04.015 20619500

[5] Kennedy M, Enander RA, Tadiri SP, Wolfe RE, Shapiro NI, Marcantonio ER. Delirium risk prediction, healthcare use and mortality of elderly adults in the emergency department. *Journal of the American Geriatrics Society*. 2014;62(3):462–469. doi: 10.1111/jgs.12692 24512171 PMC3959285

[6] Han JH, Shintani A, Eden S, et al. Delirium in the emergency department: an independent predictor of death within 6 months. *Annals of emergency medicine*. 2010;56(3):244–252. doi: 10.1016/j.annemergmed.2010.03.003 20363527 PMC3768121

[7] Fong TG, Davis D, Growdon ME, Albuquerque A, Inouye SK. The interface between delirium and dementia in elderly adults. *The Lancet Neurology*. 2015;14(8):823–832. doi: 10.1016/S1474-4422(15)00101-5 26139023 PMC4535349

[8] Stephens CE, Newcomer R, Blegen M, Miller B, Harrington C. The effects of cognitive impairment on nursing home residents’ emergency department visits and hospitalizations. *Alzheimer’s & Dementia*. 2014;10(6):835–843. doi: 10.1016/j.jalz.2014.03.010 25028060 PMC6528828

[9] Rovner BW, Casten RJ. Emergency department visits in African Americans with mild cognitive impairment and diabetes. *Journal of Diabetes and its Complications*. 2021;35(5):107905. doi: 10.1016/j.jdiacomp.2021.107905 33752964 PMC8046720

[10] Aggarwal NT, Wilson RS, Beck TL, Bienias JL, Bennett DA. Motor dysfunction in mild cognitive impairment and the risk of incident Alzheimer disease. *Arch Neurol*. 2006;63:1763–1769. doi: 10.1001/archneur.63.12.1763 17172617

[11] Vesely B, Rektor I. The contribution of white matter lesions (WML) to Parkinson’s disease cognitive impairment symptoms: A critical review of the literature. *Parkinsonism Relat Disord*. 2016;22 Suppl 1:S166–S170. 26391185 10.1016/j.parkreldis.2015.09.019

[12] Potter GG, Steffens DC. Contribution of depression to cognitive impairment and dementia in older adults. *Neurologist*. 2007;13:105–117. doi: 10.1097/01.nrl.0000252947.15389.a9 17495754

[13] Cedarbaum JM, Jaros M, Hernandez C, et al. Rationale for use of the Clinical Dementia Rating Sum of Boxes as a primary outcome measure for Alzheimer’s disease clinical trials. *Alzheimer’s & Dementia*. 2013;9(1):S45–S55. doi: 10.1016/j.jalz.2011.11.002 22658286

[14] Lu M, Pontecorvo MJ, Devous MD, et al. Aggregated tau measured by visual interpretation of flortaucipir positron emission tomography and the associated risk of clinical progression of mild cognitive impairment and Alzheimer disease: results from 2 phase III clinical trials. *JAMA Neurology*. 2021;78(4):445–453. doi: 10.1001/jamaneurol.2020.5505 33587110 PMC7885097

[15] Tible M, Sandelius Å, Höglund K, et al. Dissection of synaptic pathways through the CSF biomarkers for predicting Alzheimer disease. *Neurology*. 2020;95(8):e953–e961. doi: 10.1212/WNL.0000000000010131 32586895

[16] Cullen NC, Leuzy A, Palmqvist S, et al. Individualized prognosis of cognitive decline and dementia in mild cognitive impairment based on plasma biomarker combinations. *Nature Aging*. 2021;1(1):114–123. 10.1038/s43587-020-00003-5 37117993

[17] Iaccarino L, Chiotis K, Alongi P, et al. A cross-validation of FDG-and amyloid-PET biomarkers in mild cognitive impairment for the risk prediction to dementia due to Alzheimer’s disease in a clinical setting. *Journal of Alzheimer’s Disease*. 2017;59(2):603–614. doi: 10.3233/JAD-170158 28671117

[18] Sanchez-Catasus CA, Stormezand GN, Jan van Laar P, De Deyn PP, Alvarez Sanchez M, Dierckx RAJO. FDG-PET for prediction of AD dementia in mild cognitive impairment. A review of the state of the art with particular emphasis on the comparison with other neuroimaging modalities (MRI and perfusion SPECT). *Current Alzheimer Research*. 2017;14(2):127–142. doi: 10.2174/1567205013666160629081956 27357645

[19] Dickerson BC, Sperling RA, Hyman BT, Albert MS, Blacker D. Clinical prediction of Alzheimer disease dementia across the spectrum of mild cognitive impairment. *Archives of general psychiatry*. 2007;64(12):1443–1450. doi: 10.1001/archpsyc.64.12.1443 18056553 PMC2581771

[20] Zhu F, Li X, McGonigle D, et al.; Analyze Informant-Based Questionnaire for The Early Diagnosis of Senile Dementia Using Deep Learning. *IEEE J Transl Eng Health Med*. 2020;8:2200106. doi: 10.1109/JTEHM.2019.2959331 31966933 PMC6964964

[21] Yang YW, Hsu KC, Wei CY, Tzeng RC, Chiu PY. Operational Determination of Subjective Cognitive Decline, Mild Cognitive Impairment, and Dementia Using Sum of Boxes of the Clinical Dementia Rating Scale. *Front Aging Neurosci*. 2021;13:705782. doi: 10.3389/fnagi.2021.705782 34557083 PMC8455062

[22] Chiu PY, Tang H, Wei CY, Zhang C, Hung GU, Zhou W. NMD-12: A new machine-learning derived screening instrument to detect mild cognitive impairment and dementia. *PloS one*. 2019;14(3):e0213430. doi: 10.1371/journal.pone.0213430 30849106 PMC6407752

[23] Morris JC. The Clinical Dementia Rating (CDR): current version and scoring rules. *Neurology*. 1993;43:2412–2414. doi: 10.1212/wnl.43.11.2412-a 8232972

[24] Lin KN, Wang PN, Liu CY, Chen WT, Lee YC, Liu HC. Cutoff scores of the cognitive abilities screening instrument, Chinese version in screening of dementia. *Dement Geriatr Cogn Disord*. 2002;14:176–182. doi: 10.1159/000066024 12411759

[25] Nasreddine ZS, Phillips NA, Bedirian V, et al. The Montreal Cognitive Assessment, MoCA: a brief screening tool for mild cognitive impairment. *J Am Geriatr Soc*. 2005;53:695–699. doi: 10.1111/j.1532-5415.2005.53221.x 15817019

[26] Hung CH, Hung GU, Wei CY, Tzeng RC, Chiu PY. Function-based dementia severity assessment for vascular cognitive impairment. *Journal of the Formosan Medical Association*. 2021;120(1):533–541. doi: 10.1016/j.jfma.2020.07.001 32653387

[27] Cummings JL The Neuropsychiatric Inventory: assessing psychopathology in dementia patients. *Neurology*. 1997;48(5 Suppl 6):10S–16S. doi: 10.1212/wnl.48.5_suppl_6.10s 9153155

[28] Petersen RC, Smith GE, Waring SC, Ivnik RJ, Tangalos EG, Kokmen E. Mild cognitive impairment: clinical characterization and outcome. *Arch Neurol*. 1999;56:303–308. doi: 10.1001/archneur.56.3.303 10190820

[29] O’Bryant SE, Waring SC, Cullum CM, et al. Staging dementia using Clinical Dementia Rating Scale Sum of Boxes scores: a Texas Alzheimer’s research consortium study. *Arch Neurol*. 2008;65:1091–1095. doi: 10.1001/archneur.65.8.1091 18695059 PMC3409562

[30] O’Bryant SE, Lacritz LH, Hall J, et al. Validation of the new interpretive guidelines for the clinical dementia rating scale sum of boxes score in the national Alzheimer’s coordinating center database. *Archives of neurology*. 2010;67(6):746–749. doi: 10.1001/archneurol.2010.115 20558394 PMC2888493

[31] McKhann GM, Knopman DS, Chertkow H, et al. The diagnosis of dementia due to Alzheimer’s disease: recommendations from the National Institute on Aging and the Alzheimer’s Assocation Workgroup. *Alzheimers Dement*. 2011;7:263–269. doi: 10.1016/j.jalz.2011.03.005 21514250 PMC3312024

[32] Williams MM, Storandt M, Roe CM, Morris JC. Progression of Alzheimer’s disease as measured by Clinical Dementia Rating Sum of Boxes scores. *Alzheimer’s & Dementia*. 2013;9(1):S39–S44. 10.1016/j.jalz.2012.01.005 PMC3660405 22858530

[33] Nakata E, Kasai M, Kasuya M, et al. Combined memory and executive function tests can screen mild cognitive impairment and converters to dementia in a community: the Osaki-Tajiri project. *Neuroepidemiology*. 2009;33(2):103–110. doi: 10.1159/000222092 19494551

[34] Tschanz JT, Welsh-Bohmer KA, Lyketsos CG, et al. Conversion to dementia from mild cognitive disorder: the Cache County Study. *Neurology*. 2006;67(2):229–234. doi: 10.1212/01.wnl.0000224748.48011.84 16864813

[35] Ma F, Wu T, Miao R, Zhang W, Huang G. Conversion of mild cognitive impairment to dementia among subjects with diabetes: a population-based study of incidence and risk factors with five years of follow-up. *Journal of Alzheimer’s Disease*. 2015;43(4):1441–1449. doi: 10.3233/JAD-141566 25159674

[36] De Vriendt P, Mets T, Petrovic M, Gorus E. Discriminative power of the advanced activities of daily living (a-ADL) tool in the diagnosis of mild cognitive impairment in an older population. *International Psychogeriatrics*. 2015;27(9):1419–1427. doi: 10.1017/S1041610215000563 25901578

[37] Sugimoto T, Ono R, Kimura A, et al. Impact of Cognitive Frailty on Activities of Daily Living, Cognitive Function, and Conversion to Dementia Among Memory Clinic Patients with Mild Cognitive Impairment. *Journal of Alzheimer’s Disease*. 2020;76(3):895–903. doi: 10.3233/JAD-191135 32568192

[38] Perneczky R, Pohl C, Sorg C, et al. Complex activities of daily living in mild cognitive impairment: conceptual and diagnostic issues. *Age and ageing*. 2006;35(3):240–245. doi: 10.1093/ageing/afj054 16513677

[39] Helmer C, Stengel B, Metzger M, et al. Chronic kidney disease, cognitive decline, and incident dementia: the 3C Study. *Neurology*. 2011;77(23:2043–2051. doi: 10.1212/WNL.0b013e31823b4765 22116945

[40] Dong Y, Lee WY, Basri NA, et al. The Montreal Cognitive Assessment is superior to the Mini–Mental State Examination in detecting patients at higher risk of dementia. *International Psychogeriatrics*. 2012;24(11):1749–1755. doi: 10.1017/S1041610212001068 22687278

[41] Peters R, Beckett N, Forette F, et al. Incident dementia and blood pressure lowering in the Hypertension in the Very Elderly Trial cognitive function assessment (HYVET-COG): a double-blind, placebo controlled trial. *The Lancet Neurology*. 2008;7(8):683–689. doi: 10.1016/S1474-4422(08)70143-1 18614402

