# Supplementary material for: Emergency department visits among people with predementia highly predicts conversion to dementia
Source: PLoS One. 2022 Jun 24;17(6):e0270284. doi: 10.1371/journal.pone.0270284 (PMC9231782; doi:10.1371/journal.pone.0270284)
Supplement: S1 Table — (DOCX) [file pone.0270284.s001.docx]

**S1 Table. Comparison of demographical data between the non-converter and converter groups of the predemented participants.**

|  | EDV 0  Mean (SD) | EDV 1  Mean (SD) | EDV 2  Mean (SD) | EDV >2  Mean (SD) | *p*-value |
| --- | --- | --- | --- | --- | --- |
| N | 275 | 107 | 67 | 63 |  |
| Baseline |  |  |  |  |  |
| CDR-SB | 1.7 (1.2) | 1.8 (1.2) | 2.0 (1.5) | 1.8 (1.2) | NS |
| CASI | 77.7 (11.9) | 76.4 (13.2) | 77.3 (11.8) | 75.8 (13.2) | NS |
| HAIADL | 3.4 (2.1) | 3.9 (2.6) | 3.6 (2.2) | 3.7 (2.2) | NS |
| NPI | 4.6 (6.9) | 5.9 (7.6) | 3.9 (5.7) | 6.2 (7.2) | NS |
| MCB |  |  |  |  |  |
| CDR-SB | 3.7 (5.3) | 4.7 (5.0) | 4.1 (5.6) | 6.2 (5.6)* | 0.007 |
| CASI | -18.5 (17.7) | -20.6 (26.5) | -18.1 (27.7) | -27.7 (29.1) | NS |
| HAIADL | 7.2 (9.3) | 9.4 (9.1) | 8.9 (10.0) | 12.2 (9.6)* | 0.002 |
| NPI | 2.4 (12.1) | 1.7 (11.7) | 4.4 (10.2) | 3.0 (11.9) | NS |

# EDV, emergency department visits; CDR-SB, sum of boxes of the Clinical Dementia Rating scale; NS, non-significance; CASI, Cognitive Abilities Screening Instrument; HAIADL, History-based Artificial Intelligence Activities of Daily Living; NPI, Neuropsychiatric Inventory; MCB, Mean change from baseline.

* EDV 0= EDV 1= EDV 2< (EDV> 2)
